# New strategy to reveal the black seeds of melanomas (cancer stem cells) with their vulnerable characteristics at cellular and molecular levels

**DOI:** 10.1186/2051-1426-1-S1-P160

**Published:** 2013-11-07

**Authors:** Beatrix Kotlan, Gabriella Liszkay, Gyorgy Naszados, Zoltan Dolescall, Laszlo Toth, Laszlo Gobor, Istvan N Vamosy, Andras Szollar, Szabolcs Horvath, Klara Eles, Orsolya Csuka, Miklos Kasler, Maria Godeny, Francesco M Marincola

**Affiliations:** 1 Molecular Immunology and Toxicology, National Institute of Oncology, Budapest, Hungary; 2 Oncodermatology, National Institute of Oncology, Budapest, Hungary; 3 Radiological Diagnostics, National Institute of Oncology, Budapest, Hungary; 4 Pathogenetics, National Institute of Oncology, Budapest, Hungary; 5 Oncosurgery, National Institute of Oncology, Budapest, Hungary; 6 Surgical and Molecular Tumorpathology, National Institute of Oncology, Budapest, Hungary; 7 Board of Directors, National Institute of Oncology, Budapest, Hungary; 8 Univ Med Pharm, Tirgu Mures, Romania; 9 SIDRA Medical and Research Center, Doha, Qatar

## Objectives

A suitable approach to select cancer stem cells (CSC), the black seed of melanomas would enable their characterization and elimination.

## Methods

Cancerous tissues from primary and metastatic lesions of patients with malignant melanomas (n=150) were investigated by cell cultures and molecular genetics. Double labelled cells were sorted by BD FACSAvia Sorter. Gene expression analysis by Real Time PCR (MYiQTM, BIO-RAD) and RNA microarray (Agilent) has been performed (n=48).

## Results

**Figure 1:**
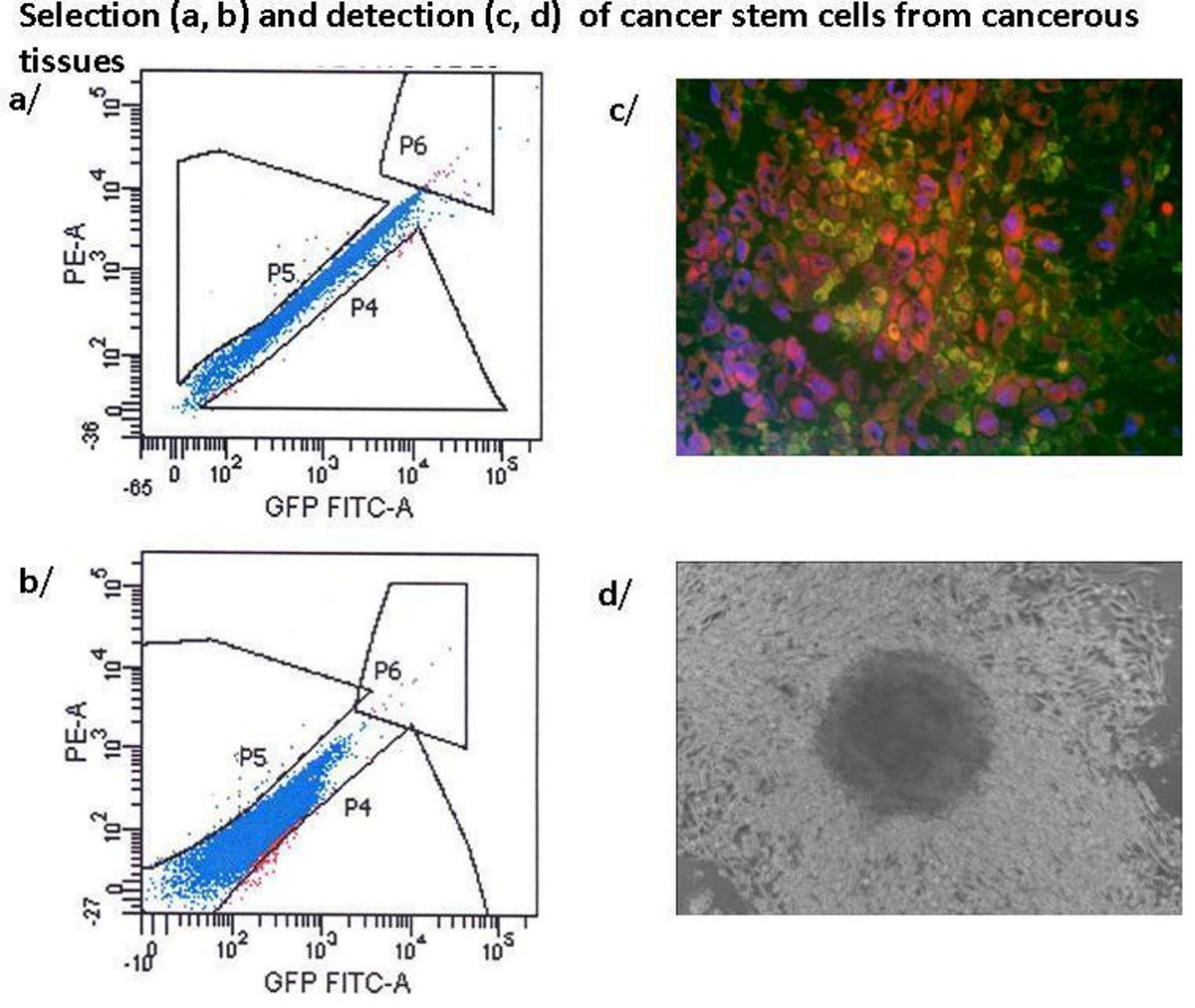


90% of the cell cultures grew and cancer initiating cells could be IF FACS sorted (0,1% - 1 %). Colocalisation of unique GD3 sialilated glycosphingolipids and antiCD20 binding was proved. Characteristic growth pattern, spheroid forming, CSC markers (e.g. CD133, Nestin, ABCB5, CD20 and unique GD3) was observed (Figure 1.a,b,c,d). We found enhanced gene expression of CXCR4 and other markers correlated to metastatic potential, clinical outcome and CSC presence. High throughput gene expression microarray analysis of RNA preparations of punch biopsies and CSC outgrowth are compared.

## Conclusion

Unique GD3 sialilated glycosphingolipids with colocalised CD20 proved to be selection markers for CSC in metastatic melanomas. Our strategy paves the way for detection and characterization of cancer stem cells and provides material for therapeutic developments to eliminate the black seeds of melanomas.

